# Effects of 3 Tesla magnetic resonance imaging exposure on the behavior and orientation of homing pigeons *Columba livia domestica*

**DOI:** 10.1371/journal.pone.0241280

**Published:** 2020-12-18

**Authors:** Daniel García Párraga, Peter L. Tyack, Vicente Marco-Cabedo, José Luis Crespo-Picazo, Xavier Manteca, Luis Martí-Bonmatí

**Affiliations:** 1 Research Department, Fundación Oceanogràfic de la Comunidad Valenciana, Valencia, Spain; 2 Biology Department, Avanqua-Oceanográfic SL, Valencia, Spain; 3 Sea Mammal Research Unit, Scottish Oceans Institute, School of Biology, University of St Andrews, East Sands, St Andrews, United Kingdom; 4 School of Veterinary Science, Universitat Autònoma de Barcelona, Barcelona, Spain; 5 Medical Imaging Department and Biomedical Imaging Research Group at La Fe University and Polytechnic Hospital and Health Research Institute, Valencia, Spain; Universitatsklinikum Wurzburg, GERMANY

## Abstract

Homing pigeons (*Columba livia domestica*) were used to test whether clinical magnetic resonance (MR) imaging disrupts orientation of animals that sense the earth’s magnetic field. Thirty young pigeons were randomly separated into three groups (n = 10/group). Two groups were anaesthetized and exposed to either a constant (no sequence) or a varying (gradient echo and echo planar sequences) magnetic field within a 3 Tesla MR unit for 15 minutes. The control group was not exposed to the MR field but shared all other aspects of the procedure. One day later, animals were released from a site they had never visited, 15 km from the home loft. Three weeks after the procedure, animals were released from a different unfamiliar site 30 km from the loft. Measured variables included the time to disappear from sight (seconds), vanishing bearing (angle), and the time interval from release to entering the home loft (hours). On first release, the group exposed to varying field gradients during image acquisition using 2 different standard sequences showed more variability in the vanishing bearing compared to the other groups (p = 0.0003 compared to control group), suggesting interference with orientation. Other measures did not show significant differences between groups. On second release, there were no significant differences between groups. Our results on homing pigeons show that regular clinical MR imaging exposure may temporarily affect the orientation of species that have magnetoreception capabilities. If exposure to MR imaging disrupted processes that are not specific to magnetoreception, then it may affect other species and other capabilities as well.

## Introduction

Many species, including bacteria, insects, molluscs, crustaceans, fish, turtles, birds, and mammals, use the earth’s geomagnetic field for navigation [1, 2]. Experimental exposure to magnetic fields has been shown to alter the orientation of a diverse array of animal species: mole-rats [3], sharks [4], honeybees [5], sea turtles [6], cockroaches [7], birds [8]. Clinical magnetic resonance (MR) imaging uses strong magnetic fields (~3 Tesla or 3T) about 60,000 times the average strength of the earth’s magnetic field (~50 μT), but the short and long-term effects of MR imaging have not been demonstrated for any animal species. Clinical MR is increasingly used for wildlife medicine in zoological institutions and rehabilitation centers, which raises concern about the effect of MR on wildlife [9].

MR exposure is considered a physiologically safe procedure in human medicine without deleterious effects at standard field strengths up to at least 3T [10–13]. However, laboratory mice exposed to a 5T constant magnetic field for 24 and 48 h ate and drank less, leading to a reduced body weight [14]. Some authors have pointed out that MR-related forces on magnetite particles located in tissues could cause injury [15]. Loggerhead sea turtles (*Caretta caretta*) exposed to MR imaging for diagnostic purposes react to the presence of varying magnetic field gradients despite being in a deep anesthetic state [16, 17, and personal observations of the authors]. During MR image acquisitions, sea turtles responded by increasing their heart rate and moving their body parts, such as the head and flippers, moving enough to impede the acquisition of quality images.

In this study, we aimed to evaluate the extent to which a short exposure to powerful magnetic fields, such as the ones used in clinical MR, can induce after-effects on normal orientation behavior. The homing pigeon (*Columba livia domestica*) was selected as a model species as they use the geomagnetic field for orientation with a highly predictable homing behavior [18, 19].

## Materials and methods

### Study birds

The experiment was approved by the Ethical Committee of the Fundación Hospital La Fe (registration number, OEBA-1-2016). Birds were provided by a local official breeder (affiliated with the “*Real Federación Colombófila Española”* society).

Thirty juvenile homing pigeons were included in the study, between 4 and 5 months of age, with no gender determination. All individuals were kept under same living conditions, were ringed for identification, and were confirmed to be clinically healthy by a specialized veterinarian. During the first months of life the birds were allowed to fly outside of the loft for several hours each day to develop their flight muscles, learn to form flocks, and learn landmarks near the loft. The animals had not participated in any previous release or experiment. We think that the daily exercise flights from the loft were unlikely to have given the young subjects close-up experience with landmarks visible from the release sites, which were 15 and 30 km away. The animals were returned to their owner once the study was finished.

### MR methodology

All pigeons underwent a 2-hour fasting period inside two transport crates (certified by the Homing Pigeon Federation; 100x75x30 cm, each with a capacity of holding 20 pigeons but only used to hold 15 animals), after which they were taken to the MR facility (84.2 km from the loft). The pigeons were then weighed (average weight 350 g) and anesthetized using a mixture of intramuscular ketamine (30 mg/kg) and diazepam (1mg/kg). The 30 anesthetized pigeons were randomly divided into three groups of 10 pigeons each. No relevant differences were observed in age or weight between the three groups. Warm water bottles were used to prevent hypothermia of anesthetized birds in the cool scanning area. The control group was kept in the preparation room and was not exposed to the MR field. The constant field group was exposed for 15 minutes to the main MR magnetic field of 3 Tesla, all at the same time inside the scanner, without running any MR sequence. The clinical MR (Philips DS Achieva, Philips Healthcare, Andover, USA) was operated at 3T with an 80 mT/m MR gradient and a slew rate of 200 T/m/s. Pigeons from the varying field group were placed simultaneously inside the MR gantry for 15 minutes, while 2 sequences were performed: a 3D Gradient Echo (TR = 9.2 ms; TE = 1.2 ms; 150 slices; gradients = 4.5 mT/m; scan time = 3:28 min) and a 2D Echo Planar (TR = 13556 ms; TE = 40 ms; 76 slices and 40 dynamics; gradients = 0.5 mT/m; scan time = 9:15 min).

All pigeons were allowed to recover from the anesthesia in an adequately heated room. The recovery time of each individual was recorded, and no problems were observed during recovery. One hour after recovery, the animals were placed back in the transport crate with free access to food and water. They stayed at the animal research facilities until the next day for their first release.

### Pigeon releases

Two controlled releases, separated by 22 days in time and 14.2 km in distance, were conducted to assess speed and accuracy of orientation behavior 1 day and 23 days after the MR exposure. Both releases were done on clear days with no overcast and at dawn to avoid exposing animals to excessive heat. The first release site (40°3’46.97”N; 0°7’24.30”W), was located 15.4 km away from the loft (39°59’0.31”N; 0°16’17.59”W) and the bearing to home from release site 1 was 235 degrees. The second release site (40°10’5.43”N; 0°1’19.04”W) was 29.6 km away from the loft, with a home bearing of 226 degrees. The distances were selected to be far enough that the birds could not find the loft by chance and not so far that the birds would get tired due to their lack of experience with long flights and to minimize the risk of predatory attacks. The environmental conditions during the first and second releases were as follows: minimum/maximum temperature 20.3/31.6°C vs. 21.8/36.1°C, and relative humidity 61% vs. 47%, respectively.

Each bird was released individually; no pigeon was released until the previous one had been out of sight for at least 5 minutes to prevent them from following each other. Pigeons from the 3 groups were alternated in the release sequence. Each pigeon was observed with binoculars (Nikon Monarch 7, 8x42) out to a maximum visible distance of approximately 2 km. Each pigeon was observed circling around the release site and then adopting a straight route of flight until it was no longer visible, at which point the compass angle of its vanishing bearing was noted. The exact time of release and time when each subject vanished from sight was recorded. As pigeons were individually identified with a microchipped ring, a reader at the entrance of the loft (TIPES^®^ MC2100) was used to register the exact time each pigeon entered the home loft. The reader was removed 72 h after release.

Finally, 3 response parameters were recorded from each subject: the duration from release to the time the subject vanished from sight flying in a straight line; the compass angle of its vanishing bearing; and the amount of time it took from release to entering the loft. Circular statistics were performed using the circular package of R-Studio [20], except for two statistics. The calculation of the 95% confidence interval of the mean vectors for vanishing bearing was made using the circ_mean function of the circular statistics toolbox for MATLAB [21]. The logic of the statistical test comparing the spread of angular values (circular analogue of homoscedasticity) for each pair of conditions was taken from [22], where spread is calculated by deviation angles that compare each measurement to the mean vector for that condition. The statistical test used for this was the Kruskal-Wallis multiple comparison (using R program kruskal.test; p-values for release 1 were adjusted with the Benjamini-Hochberg method [23] derived from [24] using R program dunnTest). The probability that each of the 3 groups had a mean vanishing bearing significantly different from the home direction was calculated using SpecMeanTestBoot function test from [20, section 5.3.3]. The probability that the 3 groups share a common mean direction was calculated using YGTestBoot from section 7.2.2 of [20]. All of the non-circular data showed significant deviations from a normal distribution judging by Q-Q plots and the Shapiro-Wilk normality test (using R program shapiro.test). Therefore, non-circular data were log-transformed and evaluated again for deviation from normality. For parameters where the log-transformed non-circular data still deviated significantly from a normal distribution (Release 1 Vanishing Time; Release 2 Vanishing Time, and Release 2 Arrival Time), differences between the 3 groups were tested using the Kruskal-Wallis test using R program kruskal.test; otherwise (Release 1 Arrival Time) differences were tested by an ANOVA using R program aov.test.

## Results

After the MR exposure, all the pigeons recovered well from anesthesia over 30–45 minutes (with no statistical differences in recovery time between groups), and no unusual behavior was observed. None of the birds died during the experimental procedures at the MR site. Table 1 shows the mean values for the variables measured from the 3 groups at both releases.

**Table 1:** Summary of homing data for the different groups.

**Release from first site (15.4 km away from the loft with a bearing to home of 235 degrees).**								
**Group**	**Vanishing Bearings**		**Vanishing Time**		**Time interval from release to entering the loft**		**# Birds Returned**	
	**Mean Vector Angle** ±**95% Confidence Interval (deg)**	**Mean Vector Length**	**Seconds**	**N**	**Average Entering Time (±SD) (Hours:Minutes)**	**N**	**>24 hours**	**Did not return**
**Control**	238±6	0.99	147±69	10	26:09±28:43	10	4	0
**Constant field**	238±11	0.96	145±80	10	14:22±21:27	10	1	0
**Varying field**	261±26	0.85	138±41	10	13:57±13:42	9	3	1
**p-value**	0.22		0.93		0.58			
**Release from second site (29.6 km away from the loft with a bearing to home of 226 degrees).**								
**Group**	**Vanishing Bearings**		**Vanishing Time**		**Time interval from release to entering the loft**		**# Birds Returned**	
	**Mean Vector Angle** ±**95% Confidence Interval (deg)**	**Mean Vector Length**	**Seconds**	**N**	**Average Entering Time (±SD) (Hours:Minutes)**	**N**	**>24 hours**	**Did not return**
**Control**	194±18	0.91	151±87	10	7:27±5:05	10	0	0
**Constant field**	206±19	0.90	174±154	10	8:22±10:01	10	1	0
**Varying field**	189±12	0.96	111±22	9	11:22±10:33	8	1	1
**p-value**	0.25		0.40		0.60			

The mean vector angle ± the 95% confidence interval and mean vector length of the vanishing bearings are given in columns 2–4 respectively. The probability that the 3 groups share a common mean direction is given in column 2 of the p-value row. The average (±SD) is given for non-circular data in columns 4 and 6, along with probability that the means differ across the 3 groups. Column 8 lists the number of birds that took >24 hours to return to their home loft.

### First release

**Fig 1:**
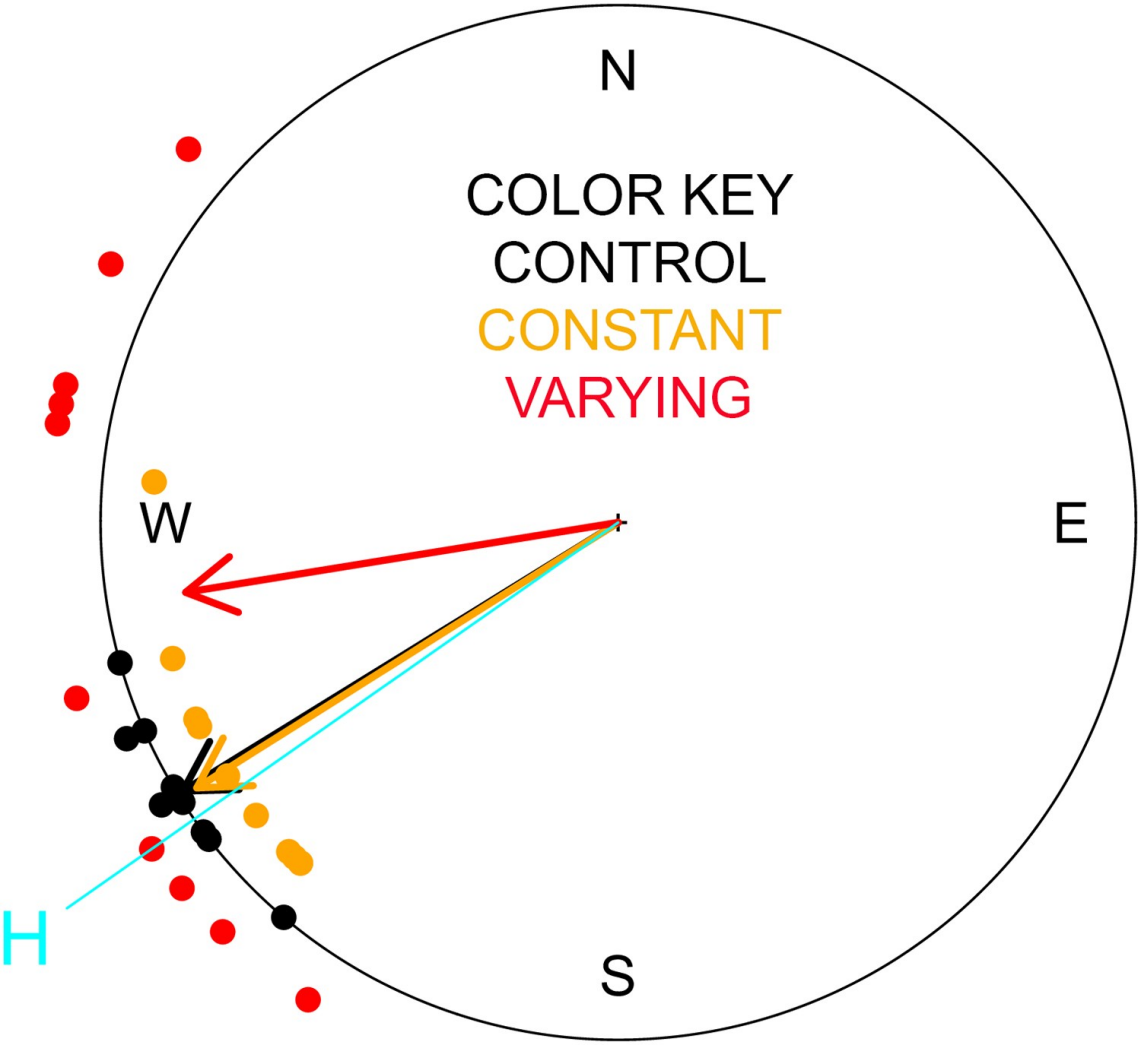
Vanishing bearings of pigeons from the first release along with the mean vector for each group. Black = control; Orange = exposed to constant magnetic field in MRI; Red = exposed to varying magnetic field from scanning sequences. Home direction is marked with the light blue line and “H”.

No differences (p = 0. 93) were observed between the 3 groups in the time each pigeon took to vanish from sight as it flew to the loft. None of the groups had a mean vanishing bearing significantly different from the home direction (Fig 1; control group, p = 0.29; constant field group, p = 0.61), although the varying field group had a close-to-significant difference from the home direction (p = 0.07). None of the groups had a mean vanishing bearing significantly different from each other (Table 1 for the comparison of all 3 groups; Table 2 for pairwise comparisons of groups), although there was a trend for the varying field group to be more different than the others on the first release.

**Table 2:** Results of testing difference in mean vanishing bearings after first and second releases between pairs of experimental conditions, using the YgTestBoot function from [20].

Condition 1	Condition 2	Release	Statistic	p-value
Control	Constant field	1	0.008	0.93
		2	1.27	0.29
Control	Varying field	1	3.79	0.11
		2	0.284	0.62
Constant field	Varying field	1	3.52	0.11
		2	2.94	0.12

The p-values from this test indicate the probability that the two groups share a common mean direction.

The deviation angles from the vanishing bearings to the mean bearing for each group showed a significantly higher scatter for pigeons in the varying field condition compared to the constant field (p = 0.01) and the control group (p = 0.0003) (Fig 1; Table 3). The difference in scatter between the constant field exposure and the control group was not statistically significant (p = 0.20).

**Table 3:** Statistical test comparing the spread of vanishing bearings (circular analog of homoscedasticity) for each pair of conditions in Release 1 (analysis from [22]).

Comparison	Z	P_unadjusted_	P_adjusted_
Constant—Control	1.30	0.20	0.20
Constant—Varying	-2.59	0.01	0.01
Control—Varying	-3.89	0.0001	0.0003

Spread is calculated by the absolute value of deviation angles from each measurement with the mean vector for that condition. The statistical test used was the Kruskal-Wallis multiple comparison (using R program Kruskal.test; p-values adjusted with the Benjamini-Hochberg method [23] using R program dunnTest). The p-values indicate the probability that the two groups share the same spread of vanishing bearings.

Finally, no differences were observed in the total time it took each pigeon to enter the loft (p = 0.58). After the first release, one pigeon from the varying group failed to return to the loft; it was probably lost and/or killed by a predator. Two of the control birds arrived after the microchip reader was removed 72 hours after release; and one of the constant field birds was found at another loft and was returned to its home loft >72 hours after release; the arrival time for these birds was listed as 72 h for statistical analyses.

### Second release

**Fig 2:**
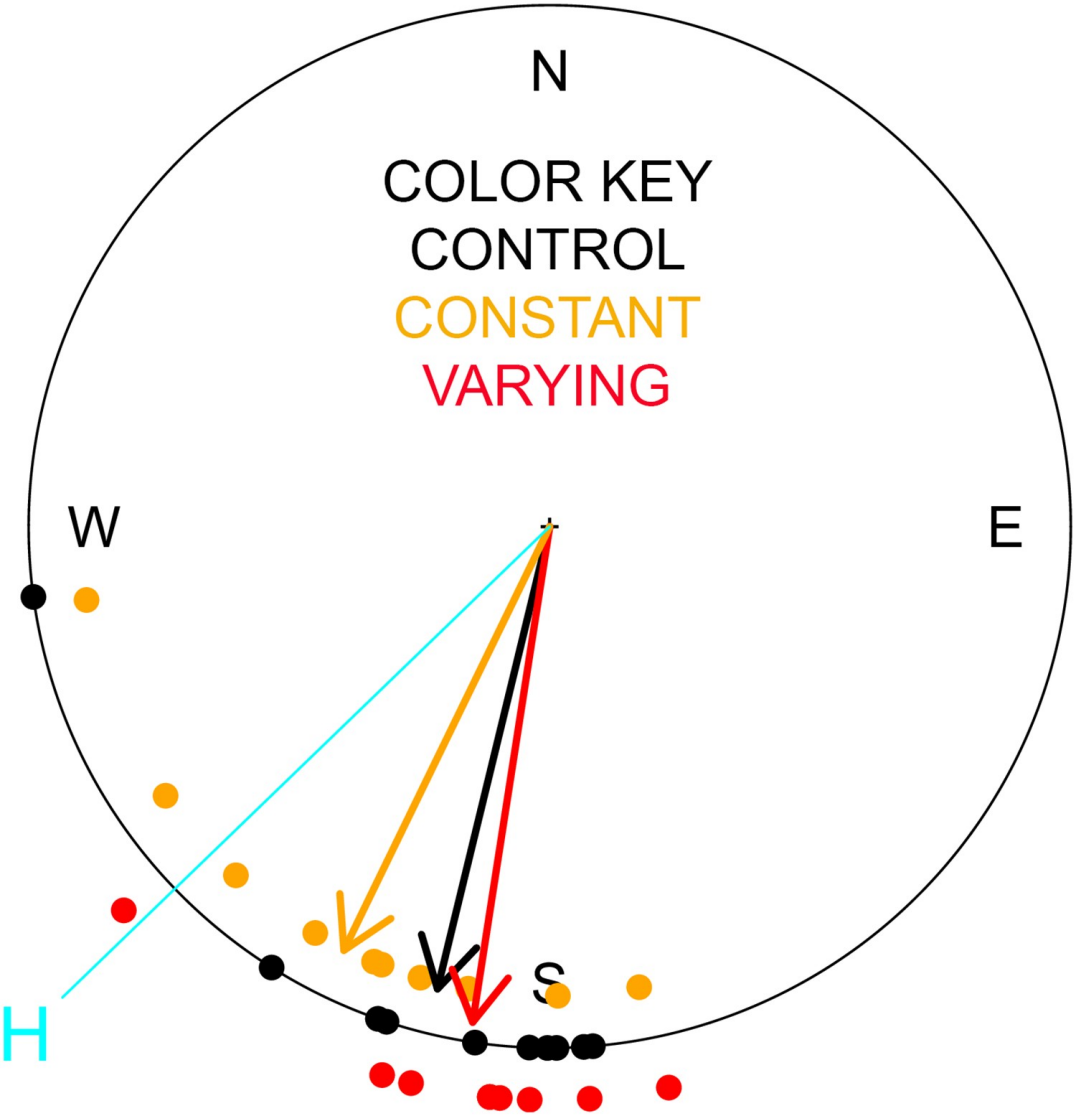
Vanishing bearings of pigeons from the second release along with the mean vector for each group. Black = control; Orange = exposed to constant magnetic field in MRI; Red = exposed to varying magnetic field from scanning sequences. Home direction is marked with the light blue line and “H”.

Fig 2 shows the vanishing bearings for birds during the second release, 23 days after exposure to all 3 conditions. No significant differences were detected between groups in the following parameters evaluated: difference in mean vanishing bearing between pairs of groups (see Table 2), dispersion of vanishing bearings with respect to the mean angle for each group (Kruskal-Wallace test across all 3 groups p = 0.30), time from release to vanishing as the pigeon headed back home (p = 0.40), mean duration from release to entering the loft (p = 0.60). However, all three groups showed a significant difference between the mean vanishing bearing and the bearing to the loft (control p = 0.002; constant field p = 0.046; varying field p = 0.0005). The fastest pigeon in this case took 70 minutes to arrive and 26 out of the 29 arrived within the first 24h. One animal from the varying field group failed to return in this second release.

## Discussion

After exposure to the varying magnetic field from standard MR acquisition sequences in a 3T MRI, the exposed pigeons showed a significant increase in the scatter of their vanishing bearings. The mean bearing for the group exposed to the varying magnetic field was farther from the correct direction angle of departure towards the pigeon loft than those of the control group and the group exposed to a constant field. The difference between the mean bearing for the varying field condition group and the home direction was not significant, but had a trend at the p = 0.07 level. The pigeons exposed to the constant magnetic field did not show any significant differences in behavior with respect to the controls in any of the response parameters that we measured.

To our knowledge this is the first demonstration of deficits in orientation after exposure to magnetic fields used for clinical diagnostic purposes in animals that use geomagnetism for orientation. Despite the increased scatter in orientation of the pigeons exposed to the varying fields, they could overcome this adverse effect and all but one pigeon returned to the loft within thirty five hours of release. Surprisingly, even though the pigeons exposed to the acquisition sequences came out more scattered, they entered the pigeon loft (average time interval from release to entering the loft) earlier. These latter results suggest that while exposure to the varying field affected orientation behavior immediately upon release, it did not have a strong effect on overall homing behavior.

There was higher scatter and more deviation of the mean vector from the home bearing for birds in all three conditions during the second release compared to control and constant exposure birds in the first release, indicating a release site bias at the more distant second site. In fact, the topography of the second release site could explain the common angle of deviation in all three groups compared to the direct line to the loft. Pigeons typically tend not to fly over mountains or forest areas but try to maintain their routes through open spaces as much as possible to minimize effort and potential risk from predators. As there were forested areas and some elevation of the terrain at approximately 1.2 km distance from the second release point in a straight line to the loft, animals in all three groups may have chosen to deviate consistently some degrees to the south to avoid those areas.

The present study reveals that diagnostic MR may not be innocuous for certain animal species. If the effect stems from interference with the mechanisms that some species use to sense geomagnetism, then the effect may be limited to species, such as homing pigeons, migratory birds or sea turtles, that are able to sense geomagnetic fields. If the effect of the exposure to MR sequences is not specific to the magnetic sense, then the adverse effect on orientation found here may also apply to other consequences in animals that do not use magnetic cues for orientation. This raises concerns that clinical evaluations using MRI may cause temporary aftereffects that impair orientation and possibly other behavioral or physiological processes. Further research is needed in order to better discriminate the potential impact and the duration of deleterious effects depending on the species and the different MR protocols used.

## Supporting information

S1 Table Response data from the 3 groups of pigeons from release site 1 and release site 2. (PDF)
