# Supplementary material for: Effects of 3 Tesla magnetic resonance imaging exposure on the behavior and orientation of homing pigeons Columba livia domestica
Source: PLoS One. 2020 Dec 18;15(12):e0241280. doi: 10.1371/journal.pone.0241280 (PMC7748148; doi:10.1371/journal.pone.0241280)
Supplement: S1 Table — (PDF) [file pone.0241280.s001.pdf]

S1 Table. Response data from the 3 groups of pigeons from release site 1 and release site 2.

		First release				Second release			
Animal ID	Group	Vanishing bearing (degrees)	|Deviation angle| with respect to mean vector for condition (degrees)	Vanishing Time (seconds)	Time to arrival at loft (hours)	Vanishing bearing (degrees)	|Deviation angle| with respect to mean vector for condition (degrees)	Vanishing Time (seconds)	Time to arrival at loft (hours)
163265	control	232	6.3	132	1.47	212	18.3	87	4.20
163267	control	238	0.3	135	12.52	175	18.7	354	12.18
163290	control	233	5.3	257	32.90	188	5.7	105	1.52
163298	control	246	7.7	237	49.88	176	17.7	199	11.73
163303	control	220	18.3	65	1.32	180	13.7	93	1.87
163307	control	246	7.7	103	11.00	182	11.7	168	4.47
163309	control	238	0.3	98	>72H	199	5.3	100	1.80
163314	control	239	0.7	125	4.93	198	4.3	112	11.93
163318	control	254	15.7	230	3.40	262	68.3	218	14.10
163320	control	237	1.3	87	>72H	179	14.7	76	10.65
Mean				146.9	26.14			151.2	7.44
SD				68.8	28.72			86.7	5.09
163263	constant field	224	13.8	113	0.74	261	55.1	204	2.80
163266	constant field	231	6.8	294	>72H	196	9.9	131	5.40
163288	constant field	223	14.8	123	6.90	179	26.9	140	8.02
163291	constant field	253	15.2	91	0.64	210	4.1	95	11.48
163293	constant field	237	0.8	108	11.62	169	36.9	113	9.43
163299	constant field	244	6.2	115	4.55	222	16.1	90	1.17
163305	constant field	245	7.2	102	1.18	201	4.9	125	1.43
163312	constant field	225	12.8	90	9.85	190	15.9	110	3.35
163315	constant field	223	14.8	118	12.33	202	3.9	133	5.43
163317	constant field	275	37.2	298	23.80	235	29.1	602	35.18
Mean				145.2	14.36			174.3	8.37
SD				80.2	21.46			153.6	10.02
163262	under sequence	235	25.8	186	6.90	186	2.8	112	34.98
163268	under sequence	311	50.2	164	LOST				
163289	under sequence	297	36.2	167	11.67	186	2.8	98	11.57
163292	under sequence	224	36.8	87	3.45	176	12.8	106	1.30
163295	under sequence	284	23.2	109	26.43	168	20.8	111	10.32
163301	under sequence	252	8.8	112	1.60	182	6.8	90	11.87
163310	under sequence	213	47.8	211	34.35	197	8.2	103	7.15
163311	under sequence	230	30.8	122	33.75	185	3.8	85	12.37
163313	under sequence	280	19.2	117	5.73	228	39.2	153	LOST
163316	under sequence	282	21.2	102	1.67	194	5.2	138	1.40
Mean				137.7	13.95			110.7	11.37
SD				41.2	13.69			22.0	10.55
